# Duration of Electrically Induced Atrial Fibrillation Is Augmented by High Voltage of Stimulus with Higher Blood Pressure in Hypertensive Rats

**DOI:** 10.1155/2014/980505

**Published:** 2014-09-25

**Authors:** Tomomi Nagayama, Yoshitaka Hirooka, Akiko Chishaki, Masao Takemoto, Yasushi Mukai, Shujiro Inoue, Takuya Kishi, Kenji Sunagawa

**Affiliations:** ^1^Department of Cardiovascular Medicine, Kyushu University Graduate School of Medical Sciences, 3-1-1 Maidashi, Higashi-ku, Fukuoka 812-8582, Japan; ^2^Department of Advanced Cardiovascular Regulation and Therapeutics, Kyushu University Graduate School of Medical Sciences, 3-1-1 Maidashi, Higashi-ku, Fukuoka 812-8582, Japan; ^3^Department of Health Sciences, Kyushu University Graduate School of Medical Sciences, 3-1-1 Maidashi, Higashi-ku, Fukuoka 812-8582, Japan; ^4^Department of Advanced Therapeutics for Cardiovascular Diseases, Kyushu University Graduate School of Medical Sciences, 3-1-1 Maidashi, Higashi-ku, Fukuoka 812-8582, Japan

## Abstract

*Objective.* Many previous clinical studies have suggested that atrial fibrillation (AF) is closely associated with hypertension. However, the benefits of antihypertensive therapy on AF are still inconsistent, and it is necessary to explore the factors augmenting AF in hypertensive rats. The aim of the present study was to investigate the correlation between arterial pressure or voltage stimulus and to the duration of electrically induced AF in normotensive or hypertensive rats. *Methods.* AF was reproducibly induced by transesophageal atrial burst pacing in spontaneously hypertensive rats (SHR) and Wistar-Kyoto rats (WKY). We did the burst pacing at high (20 V) or low (5 V) voltage. *Results.* Duration of AF did not correlate with systolic blood pressure (SBP) and stimulus voltage in WKY. However, only in SHR, duration of AF with high stimulus voltage significantly correlated with SBP and was significantly longer in high than in low voltage stimulus. *Discussion and Conclusion.* Duration of AF is augmented by high voltage stimulus with higher blood pressure in SHR.

## 1. Introduction

Many previous clinical studies have suggested that atrial fibrillation (AF) is associated with various risk factors such as cardiomyopathy, aging, diabetes mellitus, metabolic syndrome, hypertension, and obstructive sleep apnea [1–3]. In particular, hypertension is closely associated with the pathogenesis of AF [4–8]. In the Framingham study, hypertension is an increased risk of developing AF (odds ratio of 1.5 in men and 1.4 in women) after adjusting for other associated conditions [4]. Hypertensive individuals have up to 42% increased risk of AF, and as many as 60% of AF patients is reported to have hypertension [5]. Furthermore, several previous studies indicated that antihypertensive medications reduce the risk of AF [6–8]. On the other hand, incidence of AF did not differ significantly between subjects randomized to active antihypertensive treatments or placebo [3]. Considering these inconsistent backgrounds, there should be other factors than arterial pressure to accelerate AF in hypertension, and it is necessary to explore the factors augmenting AF in hypertensive rats.

Recent animal studies have indicated that rapid electrical stimulation causes electrical remodeling in the intact atrium through the shortening of action potential duration, downregulation of L-type Ca^2+^ currents, and promoting the maintenance of AF [9–13]. However, the relationships among AF, stimulus frequency, and voltage have not been fully determined. Therefore, the aim of the present study was to investigate the correlation between arterial pressure or voltage and the duration of electrically induced AF in established spontaneously hypertensive rats (SHR) and normotensive Wistar-Kyoto rats (WKY). We reproducibly induced AF by transesophageal atrial burst pacing in the pentobarbital-anesthetized condition, as reported in several recent reports [12, 14–19].

## 2. Methods

### 2.1. Studies and Animals

The study protocol was reviewed and approved by the Committee on the Ethics of Animal Experiments at the Kyushu University Graduate School of Medical Sciences and conducted according to the Guidelines for Animal Experiments of Kyushu University. Experiments were performed on male SHR and WKY (14 to 24 weeks old, SLC Japan, Hamamatsu, Japan).

### 2.2. Cardiac Hemodynamic Measurements

Rats were initially anesthetized with sodium pentobarbital (50 mg/kg intraperitoneal followed by intravenous infusion at 20 mg/kg/hour), and the body temperature was maintained at 37°C. A catheter was inserted into the femoral artery to record systolic blood pressure (SBP) and heart rate (HR). Another catheter was inserted into the femoral vein to allow for intravenous drug injections. A tracheal cannula was connected to a ventilator, and the rats were artificially ventilated with setting tidal volume at 10 mL/kg and respiratory rate at 70 strokes/min. Standard surface electrocardiography (ECG) lead II was displayed and continuously monitored using a PowerLab data acquisition system (AD Instruments, Colorado Springs, CO, USA). The ECG signals were amplified 2 mV/V using an amplifier (MEG-5200, Nihon Kohden) with a high-pass filter of 0.5 Hz and a low-pass filter of 1 kHz. The analysis of ECG recording was performed for 30 seconds before AF induction.

### 2.3. AF Induction by Transesophageal Burst Pacing

A 5-French catheter electrode (Japan Lifeline, Tokyo, Japan) was inserted into the esophagus under the monitoring of an esophageal electrogram and placed to ensure constant atrial capture with the lowest threshold. The pacing pulse of induction was rectangular in shape. Voltage of stimulus was 5 V in low (captured enough) and 20 V in high voltage stimulus (approximately 1.5 times the diastolic threshold voltage), and pulse width was 6 ms. Frequency of stimulus was fixed at 12 Hz, as reported in a previous report [18]. The atrium through the esophagus was stimulated at a cycle length of 12 ms (83 Hz) for 30 seconds via the distal electrodes pair of the catheter using an electrical stimulator (SEN-7203, Nihon Kohden) and an isolator (SS-203J, Nihon Kohden) [18]. During the induction study, AP, HR, ECG, and esophageal electrogram were monitored and recorded continuously using preceding PowerLab system. Burst pacing was performed 20 consecutive times for each rat at 5-minute intervals from recovery to sinus rhythm, and voltage stimulus was alternately in high (20 V) and low (5 V) voltage stimulus. The AF duration was measured from the end of burst pacing to the first P wave detected after the rapid irregular atrial rhythm because it is difficult to recognize waveform during the pacing. AF is defined as a rapid irregular atrial rhythm with irregular R-R intervals lasting at least 2 seconds. The incidence of AF was counted as the number of times that AF continued more than 2 seconds among 20 times.

### 2.4. Statistical Analysis

All values are expressed as the mean ± SEM. The relationship between SBP and the duration of AF was examined with a linear regression analysis with Pearson correlation coefficients. An unpaired *t*-test was used to compare the duration of AF between high and low voltage stimuli or between WKY and SHR. Differences were considered significant when the *P* value was less than 0.05.

## 3. Results

**Figure 1:**
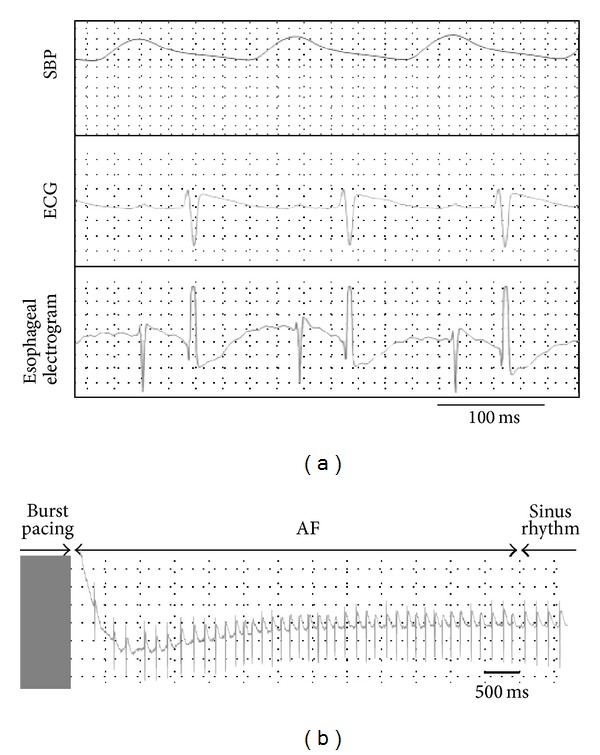
(a) A typical tracing of systolic blood pressure (SBP), ECG, and esophageal electrogram. (b) A typical time course of induced atrial fibrillation (AF) followed by burst pacing. ECG shows induced AF after burst pacing and spontaneous termination of AF.

**Figure 2:**
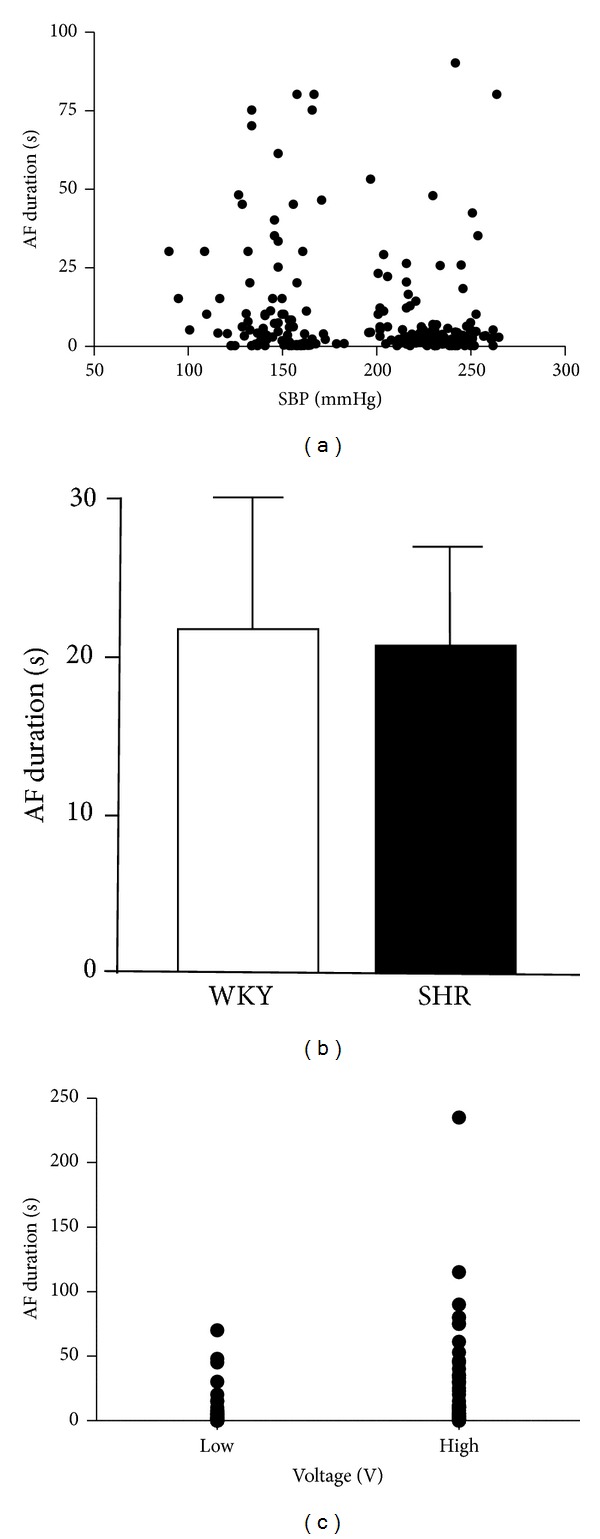
(a) Relationship between the duration of atrial fibrillation (AF) with low (5 V) and high (20 V) voltage stimulus and systolic blood pressure (SBP) in WKY and SHR. (b) The duration of AF with low (5 V) and high (20 V) voltage stimulus in WKY and SHR. (c) Duration of AF in low (5 V, low) and high (20 V, high) voltage stimulus.

**Figure 3:**
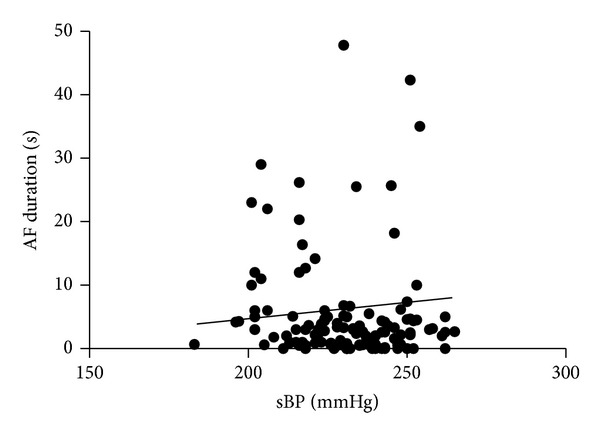
Relationship between the duration of atrial fibrillation (AF) with high (20 V) voltage stimulus and systolic blood pressure (SBP) in SHR.

**Figure 4:**
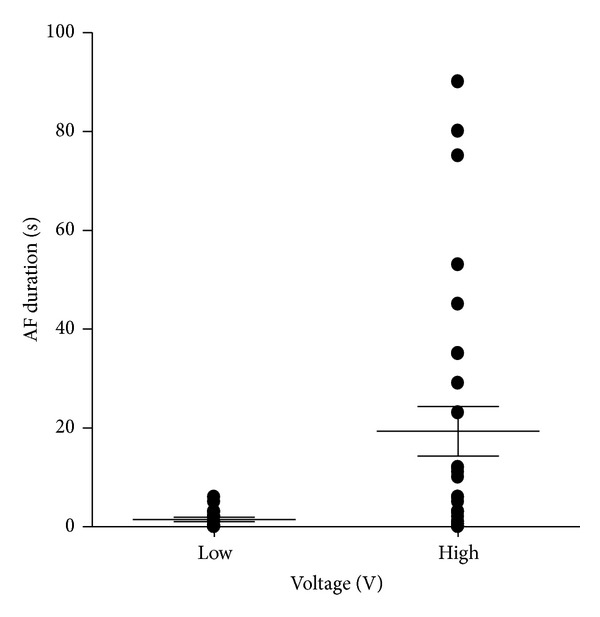
Duration of atrial fibrillation (AF) in low (5 V, low) and high (20 V, high) voltage stimuli. Horizontal lines indicate median durations.

Baseline SBP and HR were significantly higher in SHR than in WKY, and body weights did not differ between SHR and WKY. Typical SBP, ECG, and esophageal electrogram recordings before the transesophageal burst pacing are presented in Figure 1(a). Typical time course of induced AF followed by burst pacing was shown in Figure 1(b). During the stimulation, SBP did not significantly alter and ventricular arrhythmia did not occur. The inducibility of AF did not differ between SHR and WKY both in low voltage stimulus (5 V) (AF/induction; 81/130 versus 64/104 times for each 4 SHR and WKY) and in high voltage stimulus (20 V) (AF/induction; 78/118 versus 76/112 times for each 4 SHR and WKY). The duration of AF at low and high voltage did not correlate with SBP (Figure 2(a)) and not differ between WKY and SHR (Figure 2(b)). Moreover, the duration of AF did not differ between high (20 V) and low (5 V) voltage stimuli in SHR and WKY (Figure 2(c)). However, in SHR, SBP significantly correlated with the duration of AF in high voltage stimulus (20 V) (*y* = 0.0521*x* − 5.5831; *r* ^2^ = 0.47; *P* < 0.05) (Figure 3) but did not significantly correlate in low voltage stimulus (5 V). The duration of AF was significantly longer in high voltage stimulus (20 V) group than in low (5 V) group (19.3 ± 5.0 sec versus 1.5 ± 0.5 sec; *P* < 0.01) (Figure 4). In WKY, SBP did not significantly correlate with the duration of AF both in low (5 V) and in high (20 V) voltage stimuli, and the duration of AF did not differ between high voltage stimulus (20 V) and low voltage stimulus (5 V).

## 4. Discussion

Our obtained new findings were as follows: (1) duration of AF did not significantly correlate with SBP in overall of SHR and WKY, (2) SBP significantly correlated with duration of AF in SHR with high voltage stimulus, and (3) duration of AF in SHR was significantly longer in high than in low voltage stimulus. These results indicate that duration of electrically induced AF is augmented by high voltage stimulus with higher blood pressure in SHR.

Duration of AF in SHR was correlated with SBP and was significantly longer in high than in low voltage stimulus. Our motivation for the present study was that there should be other factors than arterial pressure to augmenting AF in hypertension, because of inconsistent clinical suggestions in the relationship between hypertension and AF [3–8]. Recent animal studies have suggested that electrical stimulation in rapid frequency could induce AF through shortening action potential duration and downregulation of L-type Ca^2+^currents (*I*_Ca,L_) [9–13]. In canine rapid-atrial pacing model, functional voltage-dependent ion channels were altered [20, 21]. Specifically, burst pacing is associated with significant reductions in the densities of *I*_Ca,L_, voltage-dependent Na^+^ current (*I*_Na_), and distinct transient outward K^+^ current (*I*_to_) [20, 21]. The major trigger to the upstroke of the action potential (phase 0) is *I*_Na_. *I*_Ca,L_ are also activated by membrane depolarization and contribute to plateau phase of action potential [22]. These previous studies indicate that high voltage stimulus might influence action potential duration via changes in expression of the voltage-dependent ion channels and strongly support our results in the present study. We consider that internal and external stimulus to heart with the attenuation of the structural and/or electrophysiological remodeling in left atrium, sympathetic burst excitation, or hemodynamic disruption as additive strong stimulus are necessary to progress AF in hypertension, which would be underlying risks for AF.

The mechanisms in which high voltage stimulus with hypertension augments electrically induced AF are necessary to be clarified. As discussed above, high voltage stimulus alters action potential duration [9–13, 20–22]. In hypertension, left atrium has structural and electrophysiological remodeling [2, 5, 8]. Although we did not have the results directly indicating the structural and electrophysiological remodeling in left atrium of SHR, the structural and/or electrophysiological remodeling in left atrium accompanies the development of sustained AF strongly. In further experiments, we should evaluate structural changes and altered ion channel expression including action potential duration in SHR and determine the accelerating factors to induce AF associated with structural and/or electrophysiological remodeling in left atrium of SHR.

In contrast to our present results, several previous studies have clearly suggested that AF is augmented in hypertensive rats [10, 23, 24]. Although there are methodological differences between the previous studies and the present study, we could not exclude the possibility that the present method of the electrical stimulation is too strong for WKY. Because our aim of the present study was to induce AF in acute experiments, we decided to apply the present method of the transesophageal electrical stimulation in accordance with preliminary studies, and we determined that AF was induced without hemodynamic collapse or ventricular arrhythmia. However, we also consider that our results could not provide the clinical aspects in physiological condition.

There are some limitations and unsolved issues in the present study. First, we used 14- to 24-week old rats, and we could not determine the relationship between aging and AF in the present study. Second, because we examined in acute and electrically stimulating experiments under anesthetized condition, our results could not be directly implicated to the clinical aspects. However, it is also true that hypertension is not the most specific and unique associated factor in AF, and we consider that our results could contribute to the future clinical studies. Third, we could not exclude the possibilities in which electrical stimulus-induced autonomic nervous system would affect the duration of AF. To resolve these issues, further studies stimulating electrically and recording ECG with isolated baroreflex and cut of vagal nerve in chronic and conscious state are needed. At last, we could not determine the relationship between AF and sympathetic nerve activity, because we did not measure sympathetic nerve activity. In future experiments, it is needed to examine the relationship between sympathetic nerve activity and AF in conscious state.

## 5. Conclusions

We showed that duration of electrically induced AF is augmented by voltage of stimulus with higher blood pressure in SHR. From these results, we consider that internal and external stimulus to heart with the attenuation of the structural and/or electrophysiological remodeling in left atrium, sympathetic burst excitation, or hemodynamic disruption added to hypertension would exaggerate AF.
